# Current Trends in Revision Surgery After Breast Reconstruction in China: Insights from a Nationwide Cross-Sectional Survey

**DOI:** 10.1007/s00266-025-05490-8

**Published:** 2025-12-05

**Authors:** Yi Zhang, Qi Zhang, Yiding Chen, Shuang Hao, Benlong Yang, Yingying Zhang, Xuliren Wang, Zhibo Shao, Han Zhu, Bingqiu Xiu, Jiong Wu

**Affiliations:** 1 Department of Breast Surgery, Key Laboratory of Breast Cancer in Shanghai, Fudan University Shanghai Cancer Center, Shanghai, 200032 China; 2 Department of Oncology, Shanghai Medical College, Fudan University, Shanghai, 200032 China; 3 Department of Breast Surgery, The Second Affiliated Hospital, Zhejiang University School of Medicine, Hangzhou, China; 4 Cancer Institute (Key Laboratory of Cancer Prevention and Intervention, China National Ministry of Education), the Second Affiliated Hospital, Zhejiang University School of Medicine, Hangzhou, China; 5 Human Phenome Institute, Fudan University, Shanghai, 200433 China

**Keywords:** Revision surgery, National-wide cross-sectional study, Nipple-areola complex reconstruction, Autologous fat grafting, Contralateral breast symmetry surgery

## Abstract

**Introduction:**

Revision surgery is an integral component of post-mastectomy breast reconstruction, aimed at enhancing breast aesthetics and improving patient satisfaction. While developed countries have established high rates of revision surgery, its implementation in China remains limited. We aimed to evaluate the current practices and challenges of revision surgery in Mainland China using a nationwide cross-sectional survey.

**Methods:**

A nationwide questionnaire authorized by the Chinese Anti-Cancer Association and related committees was distributed to 215 hospitals in China performing over 200 annual breast cancer surgeries, with 198 hospitals completed the survey. Data on nipple-areola complex reconstruction (NAR), autologous fat grafting (AFG), and contralateral breast symmetry surgery were collected and analyzed.

**Results:**

Of the 198 surveyed hospitals, 23.2% performed NAR, 17.2% conducted AFG, and 26.8% carried out contralateral breast symmetry surgery, with only 6.6% providing all three. (1) NAR: Both the time intervals between primary breast reconstruction and NAR and the diverse techniques of NAR were to note. The use of autologous or allogenic implants has proven to improve long-term nipple projection (median nipple shrinkage rate 5.5% vs 30%; *P *< 0.001), but its usage was still limited. (2) AFG: AFG has wide application in breast reconstruction, with the most common use remaining the correction of deformities after lumpectomy (28/34, 82.4%) or breast reconstruction after mastectomy (21/34, 61.8%). (3) Contralateral breast symmetry surgery: Contralateral breast symmetry surgery was widely accepted by patients, and a simultaneous procedure was more favored (median 80% vs 20%; *P *< 0.001).

**Conclusion:**

The implementation of revision surgery in China remains insufficient and unevenly distributed. Targeted efforts to expand specialized training, especially in plastic surgery, improve resource allocation, and adopt advanced techniques are essential.

**Level of Evidence IV:**

This journal requires that authors assign a level of evidence to each article. For a full description of these Evidence-Based Medicine ratings, please refer to the Table of Contents or the online Instructions to Authors www.springer.com/00266.

**Supplementary Information:**

The online version contains supplementary material available at 10.1007/s00266-025-05490-8.

## Introduction

The breast, as a secondary sexual characteristic, holds profound significance for breast cancer patients, both physically and psychologically [1]. In recent years, advancements in breast surgery and its interdisciplinary integration with plastic and reconstructive surgery have elevated the clinical focus on post-mastectomy breast reconstruction and revision procedures [2]. For breast cancer patients, priorities have extended beyond clinical outcomes to encompass improvements in postoperative quality of life, aesthetic restoration, and overall satisfaction, reflecting a more holistic approach to survivorship care [3].

Following breast reconstruction, patients often face challenges such as nipple loss and asymmetry between the reconstructed and contralateral breasts. Achieving bilateral symmetry requires further refinement of both the reconstructed and contralateral breasts [4, 5]. This may involve procedures such as nipple-areola complex reconstruction (NAR), autologous fat grafting (AFG), contralateral breast surgeries (e.g., augmentation, reduction, or mastopexy), and other revision surgeries aimed at enhancing aesthetic outcomes and improving patient satisfaction [5].

Globally, the demand for revision surgery is substantial, as evidenced by international studies [6]. A retrospective study in Australia found that nearly one-third (151/540) of patients undergoing breast reconstruction required at least one type of revision surgery [7]. The need was higher in implant-based breast reconstruction compared to autologous reconstruction, with post-reconstruction radiotherapy further increasing revision rates [7, 8]. Another study reported that up to 40% of patients underwent revision surgery [9]. Although a gap still exists compared to developed countries, China is steadily narrowing the disparity in this field.

To gain a deeper understanding of the current state of revisional surgery for breast cancer patients in China, anticipate future trends, and identify key barriers to their implementation, we conducted a nationwide questionnaire-based survey. In this article, we mainly focus on three critical aspects of revisional surgery: nipple–areola complex reconstruction, autologous fat grafting, and contralateral breast surgeries. The findings are presented below.

## Materials and Methods

### Questionnaire Development

A standardized questionnaire was developed with the authorization of the Chinese Anti-Cancer Association, Committee of Breast Cancer Society (CACA-CBCS) and Chinese Society of Breast Surgery (CSBrS). The development was informed by a comprehensive review of literature, clinical guidelines, and expert consultations. The questionnaire aimed to evaluate current surgical practices in breast cancer treatment across hospitals in China.

The questionnaire consisted of seven sections: respondent and hospital characteristics, axillary lymph node surgery, breast-conserving surgery, breast reconstruction, endoscopic-assisted breast surgery, revision surgery, and lymphatic and plastic surgery. In this study, we mainly focus on revision surgery. For the revision surgery component, questions focused on three key areas: nipple–areola complex reconstruction (eight questions); autologous fat grafting (eight questions); and symmetrical surgery of the contralateral breast (seven questions). Most questions were multiple-choice to facilitate efficient data collection, with options to select either a single or multiple applicable answers. Some questions required numerical or proportional responses related to clinical practices. The English version of the questionnaire is available in Supplementary File 1.

### Participants and Survey Methods

This study did not involve patient-specific data or the use of human or animal samples. All data were collected from hospital records and physician-reported practices. Ethical approval was waived by the Board of Ethical Committee at Fudan University Shanghai Cancer Center (FUSCC).

The inclusion criterion of this study was an annual volume of at least 200 breast cancer surgeries in hospitals in 2022. Four hundred and eighty-eight hospitals across China met this threshold. Based on this population, and using a 5% margin of error, a 95% confidence level, a 50% response distribution, the minimum recommended sample size was 216. Sample selection process employed a stratified strategy based on geographic distribution (province level) and annual surgical volume to ensure a representative hospital cohort. Finally, a total of 215 hospitals selected from all provinces in China and covering a wide range of surgical volume from 200–300 cases to 3000–4000 cases in 2022 was chosen in this study.

Notifications and relevant materials were sent via email to the directors of selected centers by CACA-CBCS and CSBrS. Each participating department assigned a designated specialist to complete the questionnaire, ensuring that responses reflected the institution’s current practices. All data collection and submission processes were conducted via a web-based platform for efficiency. By March 17, 2024, 198 out of 215 invited centers had fully completed the revision surgery session of the survey, and their responses were included in the final analysis.

### Statistical Analysis

The analysis was performed using SPSS 23.0 software. Measurement data following a normal distribution were described using the mean (95% confidence interval), while non-normally distributed data were presented as the median (interquartile range, IQR). Count data and ranked data were summarized using case numbers and percentages. Comparative analysis was conducted using the *t*-test for two samples following a normal distribution, while the Wilcoxon signed-rank test was applied to paired samples not following a normal distribution. Unordered categorical data were analyzed using the Chi-square test. For pairwise comparisons among three groups, *P*-values were adjusted, with *P *< 0.017 considered statistically significant. For two-tailed comparisons, *P *< 0.05 was considered statistically significant. Statistical outputs, including counts, percentages, means, medians, and ranges, were used to summarize both categorical and continuous data, and visualizations were generated using GraphPad Prism 10.0.

### Reliability and Validity Tests

The reliability of the survey was demonstrated by a Cronbach’s *α* score of 0.772, indicating acceptable internal consistency. The validity of the scale structure was supported by exploratory factor analysis, with a Kaiser–Meyer–Olkin (KMO) score of 0.669 and statistically significant Bartlett’s test of sphericity (*χ*^2 ^= 313.058, *P *< 0.000), demonstrating sufficient inter-item correlations for further analysis.

## Results

### Overview of Survey Participants

Of the 215 hospitals invited to participate, 198 hospitals (response rate 92.1%) completed the revision surgery session of the survey and were included in further analysis. These hospitals represent a comprehensive geographic distribution (province level) across China: 6.1% (12/198) in Northeast China, 16.2% (32/198) in North China, 40.9% (81/198) in East China, 6.6% (13/198) in South China, 19.7% (39/198) in Central China, 4.0% (8/198) in Northwest China, and 6.6% (13/198) in Southwest China. Notably, this 2022 survey included more non-provincial capital city (city-level) hospitals than the 2017 survey [10] (128/198 vs 31/110). Regarding hospital specialization, the majority were general hospitals 89.9% (178/198), and the rest were oncology specialty hospitals 10.1% (20/198). Annual surgical volume for breast cancer varied among the surveyed hospitals: 27.8% (55/198) performed 200~300 surgeries, 38.4% (76/198) performed 300–500, and 33.8% (67/198) performed over 500 breast surgeries per year, reflecting a diverse range of institutional capacities. More details of the characteristics of participating hospitals are presented in Table 1.

**Table 1:** Characteristics of participating hospitals

Variable	*N* (%)
*Hospital regions*	
Northeast	12 (6.06)
North	32 (16.16)
East	81 (40.91)
South	13 (6.57)
Central	39 (19.70)
Northwest	8 (4.04)
Southwest	13 (6.57)
*Per capita GDP (1000 Yuan/year)*	
<80	91 (45.96)
80~120	34 (17.17)
>120	73 (36.87)
*General hospital*	
Yes	20 (10.10)
No	178 (89.90)
*Number of inpatient beds*	
<50	80 (40.40)
50~80	44 (22.22)
>80	74 (37.37)
*Breast surgical volume (per year)*	
<300	55 (27.78)
300~500	67 (33.84)
>500	76 (38.38)
*Number of doctors with qualifications in plastic surgery*	
<2	78 (39.39)
>4	51 (25.76)
2~4	69 (34.85)

### General Information on Revision Surgery in China

Among the 198 hospitals that completed the revision surgery section of the survey in 2022, 46 hospitals (46/198, 23.2%) performed nipple-areola complex reconstruction, 34 hospitals (34/198, 17.2%) conducted autologous fat grafting, and 53 hospitals (53/198, 26.8%) carried out contralateral breast symmetry surgery. Notably, only 13 hospitals (13/198, 6.6%) performed all three types of revision surgery.

To identify factors associated with the implementation of revision surgery, we performed Chi-square test, and the results are presented in Table 2. Overall, geographic region, economic level, and hospital type (general vs. specialized) were not significantly associated with the implementation of these procedures. Instead, breast surgery departments with a larger capacity of inpatient beds and a higher volume of annual breast cancer surgeries were more likely to carry out revision surgery (Table 2). Additionally, number of surgeons qualified in plastic surgery in the department was associated with the performing of contralateral breast symmetry surgery (*P *= 0.006) (Table 2).

**Table 2:** Factors influencing the overall implementation of revision surgery

Variable	Nipple-areola complex reconstruction *N* (%)				Autologous fat grafting *N* (%)				Symmetrical surgery *N* (%)			
	Not yet	Performing	χ^2^	*P* value	Not yet	Performing	χ^2^	*P* value	Not yet	Performing	χ^2^	*P* value
Hospital regions			9.530	0.126			0.911	0.996			7.146	0.297
Northeast	8 (5.26)	4 (8.70)			10 (6.10)	2 (5.88)			11 (7.59)	1 (1.89)		
North	25 (16.45)	7 (15.22)			27 (16.46)	5 (14.71)			25 (17.24)	7 (13.21)		
East	67 (44.08)	14 (30.43)			66 (40.24)	15 (44.12)			60 (41.38)	21 (39.62)		
South	7 (4.61)	6 (13.04)			10 (6.10)	3 (8.82)			9 (6.21)	4 (7.55)		
Central	32 (21.05)	7 (15.22)			33 (20.12)	6 (17.65)			29 (20.00)	10 (18.87)		
Northwest	5 (3.29)	3 (6.52)			7 (4.27)	1 (2.94)			4 (2.76)	4 (7.55)		
Southwest	8 (5.26)	5 (10.87)			11 (6.71)	2 (5.88)			7 (4.83)	6 (11.32)		
Per capita GDP (1000 Yuan/year)			1.18	0.555			1.20	0.549			0.34	0.845
<80	67 (73.63)	24 (26.37)			77 (84.62)	14 (15.38)			67 (73.63)	24 (26.37)		
80~120	59 (80.82)	14 (19.18)			61 (83.56)	12 (16.44)			52 (71.23)	21 (28.77)		
>120	26 (76.47)	8 (23.53)			26 (76.47)	8 (23.53)			26(76.47)	8 (23.53)		
General hospital			0.01	0.935			0.44	0.505			3.77	0.052
Yes	16 (80.00)	4 (20.00)			15 (75.00)	5 (25.00)			11 (55.00)	9 (45.00)		
No	136 (76.40)	42 (23.60)			149 (83.71)	29 (16.29)			134 (75.28)	44 (24.72)		
Number of inpatient beds			10.26	0.006			7.84	0.020			13.59	0.001
<50	67 (83.75)	13 (16.25)			66 (82.50)	14 (17.50)			66(82.50)	14(17.50)		
50~80	59 (79.73)	15 (20.27)			67 (90.54)	7 (9.46)			56(75.68)	18(24.32)		
>80	26 (59.09)	18 (40.91)			31 (70.45)	13 (29.55)			23(52.27)	21(47.73)		
Breast surgical volume (per year)			6.90	0.032			11.45	0.003			16.82	<0.001
<300	48 (87.27)	7 (12.73)			49 (89.09)	6 (10.91)			46(83.64)	9(16.36)		
300~500	59 (77.63)	17 (22.37)			68 (89.47)	8 (10.53)			62(81.58)	14(18.42)		
>500	45 (67.16)	22 (32.84)			47 (70.15)	20 (29.85)			37(55.22)	30(44.78)		
Number of doctors with qualifications in plastic surgery			5.72	0.057			5.60	0.061			10.23	0.006
<2	64 (82.05)	14 (17.95)			69 (88.46)	9 (11.54)			64(82.05)	14(17.95)		
2~4	55 (79.71)	14 (20.29)			58 (84.06)	11 (15.94)			52(75.36)	17(24.64)		
>4	33 (64.71)	18 (35.29)			37 (72.55)	14 (27.45)			29(56.86)	22(43.14)		

*N* number of hospitals. % percentage of hospitals. *GDP* gross domestic product of the located province in 2022. χ^2^ chi-square value.

In our analysis of the trends throughout time, a comparison with our 2017 survey [10] revealed that the proportion of nipple–areola complex reconstruction, autologous fat grafting and contralateral breast symmetry surgery performed in provincial capital city hospitals has remained consistent over the years, while rates in non-provincial capital city hospitals were significantly lower (Table 3).

**Table 3:** Comparison of survey results on the overall implementation of revision surgery between 2017 and 2022

	OR (95% CI)	*P*-value	Interaction *P*-value*
*Nipple-areola complex reconstruction*			
Provincial capital city hospital	0.45 (0.18, 1.09)	0.078	0.297
Non-provincial capital city hospital	0.24 (0.11, 0.52)	<0.001	
*Autologous fat grafting*			
Provincial capital city hospital	0.59 (0.24, 1.44)	0.245	0.02
Non-provincial capital city hospital	0.10 (0.03, 0.33)	<0.001	
*Symmetrical surgery of contralateral breast*			
Provincial capital city hospital	0.64 (0.27, 1.52)	0.313	0.06
Non-provincial capital city hospital	0.12 (0.05, 0.27)	<0.001	

*OR* odds, ratio. *CI* confidential interval. *, significant interaction p-value indicates that the association between survey year and procedure adoption differs between provincial capital city hospitals and non-provincial capital city hospitals

### Nipple–Areola Complex Reconstruction

**Fig. 1:**
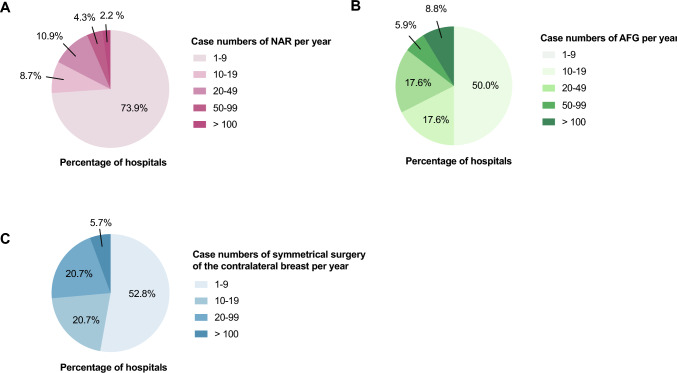
Annual case volume distribution of **A** NAR, **B** AFG, and **C** contralateral breast symmetry surgery, presented as the percentage of hospitals in each volume group

Based on the survey, nipple-areola complex reconstruction was performed in 46 out of 198 hospitals (46/198, 3.2%). The annual case volume distribution of NAR for these hospitals is shown (Fig. 1A).

In terms of surgical timing for nipple–areola complex reconstruction following breast reconstruction, the most commonly selected time interval was 6–9 months (34.8%, 16/46), followed by 3–6 months (23.9%, 11/46). Less selected intervals included over 12 months (21.7%, 10/46), 1–3 months (10.9%, 5/46), and 9–12 months (8.7%, 4/46).

Regarding surgical techniques, the utilization of rib cartilages or other implants to restore and maintain nipple projection was uncommon among the surveyed hospitals, with 67.4% (31/46) of hospitals reported either not using them or using them in less than 5% of cases. Notably, patients who underwent nipple reconstruction without implants experienced significantly greater loss of nipple projection compared to those receiving implants (median nipple shrinkage rate 30% vs 5.5%; *Z*-value = 3.855, *P *< 0.001) (Table 4). An average nipple shrinkage rate of 28.0% post-NAR surgery was reported by hospitals, regardless of implant usage. Similarly, the use of the nipple-sharing technique (contralateral nipple grafting) was limited, with 60.9% (28/46) of hospitals using this method in less than 10% of cases. Additionally, 25.6% of patients across hospitals opted for nipple–areola tattoos following NAR.

**Table 4:** Average degree of nipple shrinkage after surgery with or without the use of rib cartilage or other implants

Use of ribs or implants	Average degree of nipple shrinkage after surgery (%)		*Z*-value	*P*-value
	Median	IQR		
No	30	16.25-50	− 3.855	<0.001
Yes	5.5	0-20		

*IQR* interquartile range.

### Autologous Fat Grafting

Among the 198 hospitals, autologous fat grafting (AFG) was performed by 34 hospitals (34/198, 17.2%), with the annual case volume of AFG for these hospitals shown (Fig. 1B).

Regarding the application scope of AFG in breast reconstruction and revision, the primary use chosen by surgeons was to repair local defects after breast-conserving surgery (28/34, 82.4%), followed by improving the appearance and volume of reconstructed breasts (21/34, 61.8%). Other uses included increasing chest wall thickness before reconstruction (13/34, 38.2%) and performing total breast reconstruction (29.4%, 10/34). A smaller proportion (17.6%, 6/34) of AFG was used to correct congenital breast deformities such as Poland’s syndrome. Notably, compared to the 2017 survey, the proportion of AFG aimed at increasing chest flap thickness before breast reconstruction showed a significant increase (14.63% vs 38.24%; *χ*^2^ value = 5.47, *P *= 0.019), while other applications remained largely unchanged (Table 5).

**Table 5:** Comparison of survey results on the use of autologous fat grafting between 2017 and 2022

	2017 *N* (%)	2022 *N* (%)	*χ*^2^ value	*P*-value
Repairment of local defects after breast lumpectomy			1.38	0.241
No	12 (29.27)	6 (17.65)		
Yes	29 (70.73)	28 (82.35)		
Total breast reconstruction			0.06	0.804
No	30 (73.17)	24 (70.59)		
Yes	11 (26.83)	10 (29.41)		
Increase the thickness of chest flap before breast reconstruction surgery			5.47	0.019
No	35 (85.37)	21 (61.76)		
Yes	6 (14.63)	13 (38.24)		
Improve the appearance and volume after breast reconstruction surgery			2.38	0.123
No	9 (21.95)	13 (38.24)		
Yes	32 (78.05)	21 (61.76)		
Correction of congenital breast deformities (Poland’s Syndrome)			0.04	0.837
No	33 (80.49)	28 (82.35)		
Yes	8 (19.51)	6 (17.65)		

*N* number of hospitals; % percentage of hospitals; *χ*^2^ chi-square value.

Among hospitals performing AFG, 20.6% (7/34) used BRAVA expanders to assist in the grafting process. The use of BRAVA expanders was not associated with the number of doctors with qualifications in plastic surgery (median 5 vs 3; *Z*-value = 0.493,* P *= 0.622) (Table 6).

**Table 6:** Correlation between the use of BRAVA expanders and number of surgeons with qualifications in plastic surgery in the breast surgery department

Use of BRAVA expanders	Number of doctors with qualifications in plastic surgery		*Z*-value	*P*-value
	Median	IQR		
No	3	1-6	0.493	0.622
Yes	5	2-8		

*IQR* interquartile range.

### Contralateral Breast Symmetry Surgery

Contralateral breast symmetry surgery was performed by 53 out of 198 (26.8%) hospitals, and the annual case volume of contralateral breast symmetry surgery for these hospitals is shown in (Fig. 1C).

Among the three types of symmetry surgeries—breast augmentation, breast reduction, and breast mastopexy—contralateral breast augmentation using implants was performed significantly more frequently than breast reduction (median 6 vs 3; *Z*-value = 5.791, *P *< 0.001) and breast mastopexy (median 6 vs 3; *Z*-value = 6.047, *P *< 0.001) (Table 7).

**Table 7:** Implementation of contralateral breast symmetry surgery in 2022

Symmetrical surgery of contralateral breast	Number of cases in 2022		*Z*-value	*P*-value
	Median	IQR		
Contralateral breast augmentation	6	2-15	/	/
Contralateral breast reduction	3	1-7	5.791	<0.001*
Contralateral breast mastopexy	3	0.5-5	6.047	<0.001*

*IQR* interquartile range; *, compare to contralateral breast augmentation.

Physicians generally preferred immediate contralateral symmetry surgery over staged procedures, with the former being significantly more common (median 80% vs 20%; *Z*-value = 3.568, *P *< 0.001) (Table 8).

**Table 8:** Percentage of simultaneous or delayed symmetry procedures among all contralateral breast symmetry surgeries performed in 2022

Symmetrical surgery of contralateral breast	Percentage of cases in 2022		*Z*-value	*P*-value
	Median	IQR		
Simultaneous symmetry procedure	80	50–100	3.568	<0.001
Delayed symmetry procedure	20	0–50		

*IQR* interquartile range.

## Discussion

Revision surgery is a critical component of breast reconstruction, playing a crucial role in restoring breast aesthetics and improving patient satisfaction [4]. In this nationwide survey, the proportions of hospitals in China performing nipple-areola complex reconstruction, autologous fat grafting, and contralateral breast symmetry surgery were 23.2%, 17.2%, and 26.8%, respectively. These rates remain lower than those reported in developed countries where revision surgery is well-established [6, 7, 9, 11]: Korea (2010–2013; 38.7%, cosmetic) [12], Australia (2005–2014; 28%) [7], USA and Canada (2019; 40.2%) [9]. Based on our findings, we assumed that the limited understanding and practical skills in plastic surgery among breast surgeons in China may contribute to this disparity. Besides, low patient awareness to the need for aesthetic procedures represents another barrier. Addressing these issues is essential to improve the overall accessibility and quality of revision surgery for breast cancer patients in China.

This study also represents a series investigation building upon previous work. By including more non-provincial capital city hospitals than previous research [10], this 2022 survey provided a more comprehensive perspective on breast revision surgery practices across China. We revealed that the revision surgery rates remained consistent in provincial capital city hospitals but were significantly lower in non-provincial capital city hospitals. This disparity suggests that limited resources, infrastructure, and expertise in smaller hospitals are key barriers. Moreover, patients often prefer high-level medical centers for revision surgeries to achieve optimal aesthetic outcomes, further concentrating cases in larger institutions and exacerbating regional inequities.

### Nipple–Areola Complex Reconstruction

NAR is regarded as the final stage of breast reconstruction. According to our survey, the overall proportion of hospitals carrying out NAR in 2022 was lower than that in 2017. This decline may be attributed to the diminished demand for NAR due to the increasing adoption of nipple-sparing mastectomy (NSM). In terms of the appropriate timing of NAR, most surgeons in our study preferred to wait at least 6–9 months after primary breast reconstruction to avoid nipple malposition caused by unstable breast shape, a practice supported by global studies [13, 14]. Although immediate nipple reconstruction remains uncommon in current clinical practice, our survey identified several institutions reporting its implementation. An example is the study by Chu et al., which described a one-stage procedure using a ladder-shaped pedicled flap from the deep inferior epigastric perforator (DIEP) flap to simultaneously reconstruct both the breast and nipple [15].

Techniques for NAR include local flaps (e.g., C-V, trifoliate, and arrow flaps), grafts from distant sites (e.g., opposite nipple and earlobe), engineered tissue substitutes, or a combination with three dimensional tattooing [14]. Opposite nipple sharing, known for its natural color, texture, and projection [16], was underutilized in China according to our findings. This is probably due to concerns over donor nipple appearance, sensation, and functionality, as well as its limitation to patients with sufficient contralateral nipple tissue. Although significant advancements have been made in NAR techniques, maintaining long-term nipple projection remains a significant challenge. The use of autologous or allogenic grafts helps to overcome nipple shrinkage [17]. However, our survey found that the utilization of costal cartilage or similar techniques remains limited in China. To optimize the use of cartilage, some hospitals harvest costal cartilage during DIEP surgery for later use in nipple reconstruction 6 months postoperatively, preventing long-term projection loss [18].

### Autologous Fat Grafting

AFG is favored for its accessibility, biocompatibility, soft texture [19], and evidence based on clinical practice has demonstrated its oncological safety in breast reconstruction [20, 21]. According to our survey, the primary application of AFG in China was to correct local deformities after breast-conserving surgery, followed by improving the volume and aesthetic appearance of reconstructed breast after mastectomy. This finding demonstrated that the use of AFG to address deformities after breast-conserving surgery or breast reconstruction (either implant-based or autologous is equally important to the initial creation of an ideal reconstruction. In addition to enhancing aesthetic outcomes, the combination of implants with AFG also reduces complications such as capsular contracture and necrosis by promoting angiogenesis and improving local microcirculation [22].

Notably, the proportion of AFG used to increase the thickness of post-mastectomy chest wall before reconstruction surgery has increased significantly compared to our 2017 survey, indicating that this might be another promising clinical application of AFG. By increasing chest wall thickness and reducing skin tension, AFG facilitates subsequent prosthesis implantation, particularly in patients who have undergone post-mastectomy radiotherapy [23, 24]. Recent large-scale studies with long-term follow-up have further validated the feasibility of this approach [25].

In our survey, only a small proportion of AFG was utilized for total breast reconstruction and correction of congenital breast deformities. Historically, achieving complete breast reconstruction with AFG alone has been challenging due to difficulties in preserving fat volume and maintaining breast shape. Advancements in techniques like BRAVA-assisted fat transfer have improved outcomes, enhancing graft survival through pre-expansion of the recipient site and increased vascular density [21, 26]. Multiple successful cases of total breast reconstruction through BRAVA plus AFG have been reported [27]. According to our survey, one-fifth of hospitals in China ever used BRAVA expanders for AFG, although concerns about dermatitis and compatibility with radiation-damaged skin remain [4].

### Contralateral Breast Symmetry Surgery

As unilateral breast cancer surgery often results in breast asymmetry, contralateral symmetry surgery is frequently required to restore bilateral balance [28]. Nowadays, this contralateral symmetry procedure is widely accepted by patients. In our survey, the proportion of patients undergoing breast augmentation was higher than that of breast reduction and mastopexy. Interestingly, one study found that patients who underwent breast augmentation reported significantly higher satisfaction with their breast appearance and overall outcomes than those who received no symmetry surgery, and in contrast, no significant difference in satisfaction was observed in patients who underwent breast reduction or mastopexy [29].

Currently, the main controversy over contralateral symmetry surgery revolves around the optimal surgical timing. Our survey revealed a preference for simultaneous over staged procedures. A number of studies have demonstrated that simultaneous procedure can achieve satisfactory symmetry without increasing complication rates or interfering with subsequent therapy [28, 30, 31], even in the setting of two-stage expander-implant reconstruction [2]. Additionally, although some surgeons opted for staged procedures due to concerns that subsequent radiotherapy may cause volume changes (e.g., contracture or fibrosis), a study by Kim et al. found no significant difference in the volume ratio between irradiated and non-irradiated breasts receiving simultaneous reduction surgery at both postoperative and 18-month follow-up [32]. Nevertheless, the final decision on surgical timing is influenced by multiple factors, including the type of breast reconstruction, subsequent treatment plan, consideration of prophylactic mastectomy, and patient preference.

There are a few limitations of the present study. Firstly, the exclusion of hospitals performing fewer than 200 breast cancer surgeries per year may introduce selection basis. As in some cases, revision surgery after initial breast reconstruction was performed in plastic surgery hospitals. Secondly, although standardized questionnaires were used, specific surgical techniques of revision surgery may have varied between hospitals in ways that were not fully adjusted in analysis. Our future research will expand the sample cohort and document surgical details to enhance generalizability.

## Conclusion

In conclusion, this nationwide, hospital-based survey on revision surgery following post-mastectomy breast reconstruction has revealed the current landscape and treatment strategy preferences among surgical oncologists across China. Despite challenges in accessibility and technique utilization, especially in non-provincial capital city hospitals, the field continues to evolve in China. Key issues we discuss here include the long-term projection of nipple through NAC, the exploration of wider applications of AFG and the increasing acceptance of contralateral symmetry surgery among patients. Future efforts should focus on expanding access, training surgeons with plastic surgery skills, and advancing innovative technologies to improve aesthetic outcomes for breast cancer patients.

## Supplementary Information

Below is the link to the electronic supplementary material.

Supplementary file1 (DOCX 19 KB)
