# Supplementary material for: Current Trends in Revision Surgery After Breast Reconstruction in China: Insights from a Nationwide Cross-Sectional Survey
Source: Aesthetic Plast Surg. 2025 Dec 5;50(7):2533–42. doi: 10.1007/s00266-025-05490-8 (PMC13102828; doi:10.1007/s00266-025-05490-8)
Supplement: Supplementary file 1 — Supplementary file1 (DOCX 19 KB) [file 266_2025_5490_MOESM1_ESM.docx]

**Supplementary file 1. Questionnaire**

**Section 1: General Information About You**

**Q1. Respondent information:**

**Q1.1: Gender**

- ( ) Male
- ( ) Female

**Q1.2: Age**

- ( ) 21-30 years
- ( ) 31-40 years
- ( ) 41-50 years
- ( ) 51-60 years
- ( ) 61-70 years

**Q1.3: Professional Title**

- ( ) Resident Physician
- ( ) Attending Physician
- ( ) Associate Chief Physician
- ( ) Chief Physician

**Q1.4: Department**

- ( ) General Surgery Department
- ( ) Oncological Surgery Department
- ( ) Thyroid and Breast Surgery Department
- ( ) Breast Surgery Department

**Q1.5: Years of performing breast cancer surgery?**

- ( ) Not started
- ( ) ≤1 year
- ( ) 1-2 years
- ( ) 2-3 years
- ( ) 3-4 years
- ( ) 4-5 years
- ( ) 5-10 years
- ( ) 10-15 years
- ( ) >15 years

**Q1.6: Years of performing breast reconstruction surgery?**

- ( ) Not started
- ( ) ≤1 year
- ( ) 1-2 years
- ( ) 2-3 years
- ( ) 3-4 years
- ( ) 4-5 years
- ( ) 5-10 years
- ( ) 10-15 years
- ( ) >15 years

**Q1.7: Years of performing endoscopic breast cancer surgery +/- reconstruction surgery?**

- ( ) Not started
- ( ) ≤1 year
- ( ) 1-2 years
- ( ) 2-3 years
- ( ) 3-4 years
- ( ) 4-5 years
- ( ) 5-10 years
- ( ) 10-15 years
- ( ) >15 years

**Section 2: General Information About Your Hospital**

**Q1: General Information About Your Hospital**

**Q1.1: Hospital Name:** ________________

**Q2: Department Information**

**Q2.1: Department Name:** ________________

**Q2.2: Number of Beds:** ________________

**Q2.3: Number of Doctors Specializing in Breast Surgery (Attending Physician and Above):** ________________

**Q2.4: Number of Doctors with Plastic Surgery Licenses:** ________________

**Please provide information based on your hospital's data for the entire year of 2022.**

**Q3: Survey on Breast Cancer Surgeries in Your Department**

**Q3.1: Total number of breast cancer surgeries performed in your hospital in 2022: ______ cases.** (Exclude unplanned reoperations due to complications and secondary modifications after breast-conserving or reconstruction surgeries.)

**Section 3: Status of Revision Surgery Following Breast Reconstruction in Your Department**

**Q1: Overview of nipple-areola complex reconstruction**

**Q1.1 Does your department nipple-areola complex reconstruction?**

- ( ) Not performed [Please skip subsequent questions]
- ( ) Performed

**Q1.2 Number of nipple-areola complex reconstruction performed in 2022: ___ cases.**

**Q1.3 Average time interval between breast reconstruction and nipple-areola complex reconstruction:**

- ( ) 1-3 months
- ( ) 3-6 months
- ( ) 6-9 months
- ( ) 9-12 months
- ( ) >12 months

**Q1.4 Proportion of nipple-areola complex reconstruction using rib cartilages or other implants: _______%.**

**Q1.5 Proportion of nipple-areola complex reconstruction using nipple-sharing technique: _______%.**

**Q1.6 Average nipple shrinkage rate (without implant): _______%.**

**Q1.7 Average nipple shrinkage rate (with implant): _______%.**

**Q1.8 Proportion of patients undergoing nipple-areola complex tattooing postoperatively: _______%.**

**Q2: Overview of Autologous Fat Grafting**

**Q2.1 Does your department perform autologous fat grafting?**

- ( ) Not performed [Please skip subsequent questions]
- ( ) Performed

**Q2.2 Number of autologous fat grafting procedures performed in 2022: _______ cases.**

**Q2.3 Scope of application (multiple selection):**

- ( ) Repair local defects after breast conserving surgery
- ( ) Total breast reconstruction
- ( ) Increase chest wall thickness before reconstruction
- ( ) Improve the appearance and volume of reconstructed breasts
- ( ) Correct breast congenital deformity, such as Poland's Syndrome

**Q2.5 Is it combined with BRAVA use?**

- ( ) No
- ( ) Yes

**Q3: Overview of Contralateral Breast Symmetry Surgery**

**Q3.1 Does your department perform contralateral breast symmetry surgery?**

- ( ) Not performed [Please skip subsequent questions]
- ( ) Performed

**Q3.2 Number of contralateral breast symmetry surgery performed in 2022: _______ cases.**

**Q3.3 Among all patients receiving contralateral symmetry surgery, the proportion of performed simultaneously: _______%.**

**Q3.4 Among all patients receiving contralateral symmetry surgery, the proportion of performed in staged operations: _______%.**

**Q3.5 Among all patients receiving contralateral symmetry surgery, number of breast augmentation using implants: _______ cases.**

**Q3.7 Among all patients receiving contralateral symmetry surgery, number of breast reduction: _______ cases.**

**Q3.8 Among all patients receiving contralateral symmetry surgery, number of breast mastopexy: _______ cases.**
